# Allicin-based biomimetic nanoparticles of the erythrocyte membrane for the delivery of lumefantrine to enhance its antimalarial effect

**DOI:** 10.1016/j.ijpx.2026.100487

**Published:** 2026-01-11

**Authors:** Chuyi Yu, Xiaobo Li, Keneng Cai, Weichi Jiang, Wanying Chen, Run Xia, Mengyao Xu, Jianjia Feng, Chengli Ling, Sheng Zhou, Yinhuan Chen, Feng Zeng, Qin Xu, Xiao He, Mingqiang Li, Jianping Song, Jianming Liang

**Affiliations:** a Artemisinin Research Center, Guangzhou University of Chinese Medicine, Guangzhou 510006, China; b The First Affiliated Hospital of Guangzhou University of Chinese Medicine, Guangzhou 510405, China; c Qingyuan Polytechnic, Panlong Garden, Dongcheng Street, Qingcheng District, Qingyuan, Guangdong 511510, China; d Hunan Academy of Chinese Medicine, Yuehua Road, Yuelu District, Changsha 410013, China; e Guangdong Provincial Second Hospital of Traditional Chinese Medicine, Guangzhou 510006, China

**Keywords:** Lumefantrine, Allicin, Erythrocyte membrane biomimetic nanoparticles, Multiple pathways, *Pb*ANKA-infected malaria

## Abstract

Owing to the emergence of drug resistance and the lack of effective vaccines, malaria continues to seriously harm the physical and mental health of a multitude of individuals, warranting the need to explore new antimalarial strategies. In this study, we developed allicin-based biomimetic nanoparticles of the erythrocyte membrane (PECm-Allicin@LM) through a sonication method for the delivery of lumefantrine (LM), a hydrophobic antimalarial drug. PECm-Allicin@LM showed regular spherical morphology with a mean diameter of 120 nm and retained most of the major proteins on the erythrocyte membrane. PECm-Allicin@LM was stable and sustained the release of LM. Flow cytometry analysis showed that PECm-Allicin@LM could deliver LM to *Plasmodium*-infected erythrocytes to kill the parasite. The nanoparticles, disguised as erythrocytes, could trap merozoites and competitively inhibit them from repeatedly infecting normal erythrocytes. Allicin in the nanoparticles not only dissolved LM but also disrupted the mitochondrial function of malaria parasites, working together to combat malaria and improve the immune dysfunction caused by malaria. Giemsa staining was performed to determine the infection rate in *Pb*ANKA-infected mice. In *Plasmodium berghei* ANKA strain-infected ICR mice, PECm-Allicin@LM significantly reduced infection rates, prolonged survival time, and attenuated *Plasmodium-*induced weight loss, anemia, and organ injury. Overall, these nanoparticles combine the advantages offered by the erythrocyte membrane, allicin, and LM to effectively combat malaria, representing a new antimalarial strategy targeting multiple pathways.

## Highlights

• Allicin-based biomimetic nanoparticles of the erythrocyte membrane (PECm-Allicin@LM) for the delivery of lumefantrine (LM).
• Targeted delivery LM to *Plasmodium*-infected erythrocytes to kill the parasite.
• Trapping merozoites and competitively inhibiting them from reinfection of normal erythrocytes.
• Allicin synergistically fights against malaria and improves the immune function disruption induced by malaria infection.

## Graphical abstract

Allicin-based biomimetic nanoparticle of the erythrocyte membrane (PECm-Allicin@LM) was developed for the delivery of lumefantrine (LM). PECm-Allicin@LM showed excellent performance, suitable particle size, and good stability. Allicin present in the nanoparticles not only dissolved LM but also synergistically fought against malaria and improved the malaria infection-induced disruption in immune function, thereby executing the proverbial killing of two birds with one stone.

**Figure:**
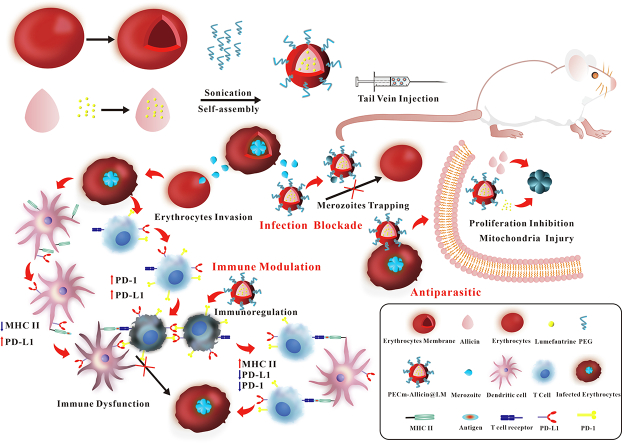

## 1 Introduction

Malaria, an insect-borne infectious disease, is one of the three deadliest infections worldwide, along with tuberculosis and acquired immunodeficiency syndrome (Makam and Matsa, 2021). It severely endangers the physical and mental health of a large number of people. The parasite strains that invade the human body include *Plasmodium falciparum*, *Plasmodium vivax*, *Plasmodium malariae*, *Plasmodium ovale*, and *Plasmodium knowlesi*, with *P. falciparum* causing the most serious disease and exerting the greatest impact on human health (Schneider and Salas, 2022). The World Health Organization estimated that there were 247 million malaria cases globally in 2021, up from 241 million in 2020, and the number of cases remains very high (World Health O, 2022a). Currently, many types of drugs, such as quinoline derivatives, artemisinin derivatives, and antifolates, are used to treat malaria. Unfortunately, almost all antimalarial drugs currently in use have records of resistance, and effective vaccines are still being explored (Walker and Rogerson, 2023). It is, therefore, necessary to explore new antimalarial strategies, for example, by optimizing existing antimalarials and discovering and developing new drugs.

Lumefantrine (LM), formerly known as benflumetol, is an antimalarial fluorene derivative belonging to the amino alcohol class of compounds that was synthesized in China in the 1970s (Olliaro and Trigg, 1995). LM exhibits powerful antimalarial activity and can be used to treat different types of malaria parasites; however, the mechanism of its action remains unclear and might involve the inhibition of DNA synthesis in parasites (Basco et al., 1998; Bin SRL et al., 2001). Although the parasite-killing effect of LM is thorough and long lasting, its onset is slow; therefore, LM is often used in combination with other drugs. Artemether–lumefantrine (Coartem®) is the most widely used artemisinin-based combination therapy for treating uncomplicated *P. falciparum* malaria globally (White et al., 1999; Groger et al., 2018; Derbie et al., 2020; World Health O, 2022b). Clinically, LM usage is limited to oral dosage forms, owing to its water-insoluble nature. Improving the water solubility of LM to enhance its dosage forms and routes of administration may overcome the shortcomings related to its slow onset and might expand the scope of its clinical application (Matshe et al., 2023).

Nanodrug delivery systems can encapsulate poorly water-soluble antimalarial drugs and stably deliver them to lesion sites, thereby increasing the effectiveness of antimalarial treatment (Rahman et al., 2019). The strategy involving the biomimicking of the erythrocyte membrane in nanodrug delivery systems can potentially capture merozoites *via* host–pathogen interactions and provide enhanced delivery of antimalarial drugs to *Plasmodium*-infected erythrocytes due to rosetting, thereby enhancing the antimalaria effect in the blood stage of infection (He et al., 2023; Zuo et al., 2022; Najer et al., 2014). Biomimetic nanoparticles of the erythrocyte membranes can be prepared by wrapping olive oil droplets with the erythrocyte membrane using a simple ultrasound method, which combines the merits of both erythrocyte membrane (specifically absorbing and neutralizing toxicants through biological binding) and olive oil droplets (nonspecifically soaking up toxicants through physical partitioning) for safe and effective detoxification of organophosphate compounds (Chen et al., 2019). The abovementioned type of cell-membrane-cloaked oil nanodrug delivery system is apparently well-suited for use against malaria, as it has biomimetic properties of the erythrocyte membrane and can carry antimalarial oil as well as an oil-soluble antimalarial drug.

Allicin, a sulfur-containing active ingredient extracted from *Allium sativum* L. bulbus, is the main active component present in garlic oil. It possesses anti-inflammatory, immunomodulatory, anticancer, antimicrobial, and antiparasitic properties, exhibiting a broad range of pharmacological activities (Borlinghaus et al., 2014; Waag et al., 2010). Allicin can cause necrotic death in *Leishmania* by inducing calcium and mitochondrial dysregulation, and partially protects the host against *Plasmodium* by enhancing innate and adaptive immune responses (Corral et al., 2016; Feng et al., 2012). Therefore, allicin may serve as an oil-based core of a cell-membrane-cloaked oil nanodrug delivery system with antimalarial effects and might dissolve other water-insoluble antimalarial drugs.

**Scheme 1:**
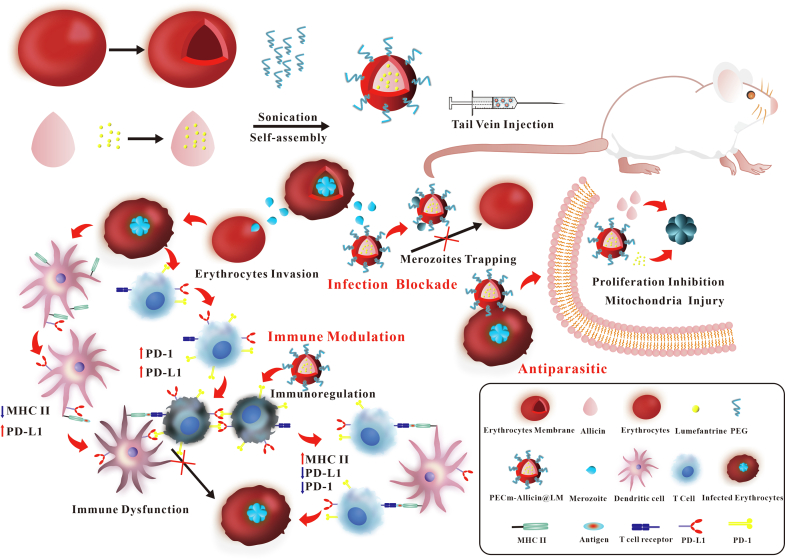
Schematic illustration of the antimalarial mechanism of PECm-Allicin@LM.

We found that LM could be dissolved in allicin. Based on the abovementioned studies and our preliminary exploration, in this study, we prepared lumefantrine-allicin-loaded erythrocyte membrane bionic nanoparticles (PECm-Allicin@LM) using allicin dissolved with LM as the oil-based core, the erythrocyte membrane as the film material, and 1,2-distearoyl-sn-glycero-3-phosphoethanolamine-poly(ethyleneglycol) (DSPE-PEG) as the surface stabilizer. We aimed to endow PECm-Allicin@LM with multimodal antimalarial ability (Scheme 1) and evaluated its *in vivo* efficacy.

## 2 Materials and methods

Phosphate-buffered saline (PBS) and Roswell Park Memorial Institute (RPMI) 1640 medium were purchased from Procell Life Science &Technology Co., Ltd. (China). Fetal bovine serum (FBS) was purchased from Gibco (USA). N-(carbonyl-methoxypolyethyleneglycol 2000)-1,2-distearoyl-sn-glycero-3-phosphoethanolamine (mPEG-DSPE, 2 K) was obtained from Hunan Huateng Pharmaceutical Co., Ltd. (China). A Giemsa staining kit was purchased from Nanjing Jiancheng Bioengineering Research Institute Co., Ltd. (China). Tween-80 was purchased from Shanghai Titan Scientific Co., Ltd. (China). The red fluorescent cell membrane dye DiD was purchased from Shanghai Titan Scientific Co., Ltd. (China). Percoll was obtained from GE Healthcare (Sweden). Lumefantrine was provided by Shanghai Aladdin Biochemical Technology Co., Ltd. (China). Lumefantrine-d9 was provided by Toronto Research Chemicals (Canada). Allicin was provided by Chengdu Pulis Biotechnology Co., Ltd. (China). 5–6(and 6)-carboxyfuorescein diacetate, succinimidyl ester (CFDA-SE) was purchased from Yeasen Biotechnology (Shanghai) Co., Ltd. (China). Hoechst 33342 (HO) was purchased from AbMole Biotechnology (Shanghai) Co., Ltd. (China). Thiazole orange (TO) was purchased from GlpBio (USA). MitoTracker™ Deep Red FM was purchased from Thermo Fisher Scientific (USA). The antibodies used for flow cytometry were from Elabscience (China).

Six- to eight-week-old male ICR mice and Sprague–Dawley (SD) rats were purchased from the Guangdong Medical Laboratory Animal Center (Guangzhou China). The *Pb*ANKA strain was a gift from Prof. Jian Li at Xiamen University, China. All animal experiments were approved by the Ethics Committee of the Institute of Science and Technology, Guangzhou University of Chinese Medicine. All animals were housed in an animal facility at the Institute of Science and Technology, Guangzhou University of Chinese Medicine, adhering to the guidelines for the protection and welfare of animal subjects.

LM was dissolved in allicin oil at 50 mg/mL, and this solution was added to the erythrocyte membrane suspension (the sample was adjusted to 1× PBS), resulting in a mixture with a membrane protein-to-oil ratio of 1:5.74 (weight/weight, *w*/w). The mixture was sonicated using a probe sonicator (Scientz-IID, 250 W; 5 s on and 5 s off) for 10 min in an ice bath. Thereafter, an appropriate amount of powdered mDSPE-PEG_2000_ was added to the mixture at a membrane protein-to-powder mDSPE-PEG_2000_ ratio of 1:1 (*w*/w), followed by sonication (250 W; 5 s on and 5 s off, 5 min). After sonication, the liquid was filtered through a 0.22 μm polycarbonate filter, and the nanoparticles thus formed were referred to as PECm-Allicin@LM. The erythrocyte membrane was isolated according to a previously reported method (He et al., 2019), and its protein content was measured using the BCA Protein Assay Kit (Beyotime Biotechnology, China).

The particles size, polydispersity index (PDI), and zeta potential of PECm-Allicin@LM were determined using the Zetasizer Nano-ZSP (Malvern Instruments, UK), and the morphological characteristics were observed using transmission electron microscopy (TEM, JEOL, Japan). To evaluate the stability of PECm-Allicin@LM, changes in particle size and PDI over 5 days at 4 °C in PBS and over 4 h at 37 °C in 10% FBS were detected using a Zetasizer Nano-ZSP.

The total protein content of the erythrocyte membrane of PECm-Allicin@LM was analyzed using sodium dodecyl sulfate-polyacrylamide gel electrophoresis (SDS-PAGE). A BCA protein assay kit was used to quantify the total protein content of the samples. The samples were mixed with 5× loading buffer (Epizyme Biotech, China) and heated at 100 °C for 10 min. Each sample was added to the gel wells after the aforementioned treatment. Protein electrophoresis was performed at a constant voltage (80 V for 30 min on the stacking gel and 120 V for 120 min on the separating gel).

A high-performance liquid chromatography (HPLC) method was used to dertemine the encapsulation rate and drug loading in PECm-Allicin@LM. To detect the concentrations of LM and allicin in PECm-Allicin@LM, PECm-Allicin@LM was diluted with methanol (1:9, *v*/v), placed in an ultrasonic bath for 3 min to disrupt the nanoparticle structure, and filtered through a 0.22 μm microfiltration membrane. The concentrations of LM and allicin were measured using HPLC (Agilent, USA), with reference to previous studies (Wu et al., 2009; Han et al., 2016), equipped with a C18 column (DIKMA, China). The mobile phase used for LM was a mixture of methanol, water, glacial acetic acid, and triethylamine (94.9:4:1:0.1, *v*/v/*v*/v); the flow rate was 1.0 mL/min, the detection wavelength was 335 nm, and the column temperature was 30 °C. For allicin, the mobile phase used was a mixture of methanol and water (85:15, v/v); the flow rate was 1.0 mL/min, the detection wavelength was 214 nm, and the column temperature was 30 °C.

The *Pb*ANKA-infected mouse model of malaria was generated as described previously (He et al., 2023). *Pb*ANKA-infected ICR mice were used as passage mice. The infection rate of *Pb*ANKA-infected mice was determined using a Giemsa Stain Kit according to the manufacturer's instructions. At an infection rate of 5–10%, 20 μL blood was collected from the orbital venous plexus of the passage mice, under isoflurane anesthesia, in a disposable micro blood collection tube. The 20 μL blood sample was diluted 100 times with PBS, and the total number of erythrocytes was counted under a microscope. The number of *Pb*ANKA-infected erythrocytes was estimated as the product of the infection rate and the total number of erythrocytes. Each ICR mouse was intraperitoneally injected with 200 μL PBS containing 1 × 10^7^ *Pb*ANKA-infected erythrocytes, and mice with simulated infection were intraperitoneally injected with an equal volume of PBS for use as the control group.

The neutralization effect of PECm-Allicin@LM on merozoites was determined according to a previous report (He et al., 2023). Briefly, blood collected from *Pb*ANKA-infected mice was centrifuged at 700 ×*g* for 5 min and washed three times with PBS. The blood cell precipitates were washed with PBS three more times and centrifuged at 3200 ×*g* for 5 min to purify total erythrocytes. The purified total erythrocytes suspended in PBS were then gently added to a 65% Percoll separation solution (1:5, *v*/v) in a 15 mL centrifuge tube and centrifuged at 2500 ×*g* for 30 min. After centrifugation, the upper layer of erythrocytes in the solution was carefully transferred to another 15 mL centrifuge tube and washed three times with PBS, and the precipitate obtained after the last centrifugation step was considered as *Pb*ANKA-infected erythrocytes.

Merozoites were extracted from *Pb*ANKA-infected erythrocytes by breaking down the membrane under hypotonic conditions. First, ultrapure water was quickly added to *Pb*ANKA-infected erythrocytes, and the suspension was mixed by swirling. The suspension was then transferred to a 2 mL EP tube and centrifuged at 800 ×*g* for 10 min; the supernatant thus obtained was discarded, and the precipitate was washed twice with ultrapure water. After the last centrifugation step, the extracted merozoites (yellow-brown precipitate) at the bottom of the EP tube were resuspended in 1× PBS containing 0.1% glucose to maintain their activity.

Merozoites were incubated with DiD-labeled nanoparticles (PECm-Allicin@DiD) in RPMI 1640 medium containing 10% FBS and 4 μmol L^−1^ HO, at 37 °C for 75 min in the dark. The mixture was then centrifuged, and the precipitate was washed three times with 1× PBS containing 0.1% glucose. Finally, the merozoites were mounted on a cell slide with glycerol jelly mounting medium and visualized using a laser confocal microscope (FV3000, Olympus).

Next, we simulated erythrocyte invasion by merozoites *in vitro* and explored the effect of PECm-Allicin@LM on this process. Merozoites and normal erythrocytes were labeled with HO and CFDA-SE respectively. The fluorescently labeled merozoites and normal erythrocytes were mixed and incubated in 1× PBS containing 0.1% glucose and different drugs (PBS, LM, Allicin, LM + Allicin, or PECm-Allicin@LM) for 30 min. The PBS group was regarded as the model group, in which merozoites invaded normal erythrocytes. After incubation, fluorescence emission from the samples was detected using flow cytometry.

DNA and RNA in *Pb*ANKA-infected erythrocytes were labeled with HO and TO, respectively, as described previously (He et al., 2023; Grimberg et al., 2008), to distinguish normal and *Pb*ANKA-infected erythrocytes, as well as *Pb*ANKA-infected erythrocytes at different stages. The total erythrocytes purified from *Pb*ANKA-infected mice and PECm-Allicin@DiD were co-incubated in RPMI 1640 medium containing 10% FBS, 4 μmol L^−1^ HO, and 100 ng/mL TO, at 37 °C for 75 min in the dark. The mixture was then centrifuged, and the precipitate was washed three times with 1× PBS. Finally, the cell precipitate was resuspended in PBS, and the uptake of nanoparticles by different erythrocytes was detected using flow cytometry. Data were analyzed using the FlowJo_V10 software, as described previously (He et al., 2023; Grimberg et al., 2008).

The purified total erythrocytes from *Pb*ANKA-infected mice were collected as described in a previous section, added equally to the RPMI 1640 medium containing 4 μmol L^−1^ HO, 100 ng/mL TO, and different drugs (PBS, LM, Allicin, LM + Allicin, or PECm-Allicin@LM), and incubated for 75 min in the dark. After washing with PBS three times, the erythrocytes were stained with MitoTracker™ Deep Red FM for 30 min. After washing three times with PBS, mitochondrial activity in *Pb*ANKA-infected erythrocytes was evaluated using flow cytometry, as described previously (He et al., 2023).

Twelve male SD rats were randomly divided into three groups and administered a single intravenous injection of LM (0.42 mg/kg), LM + Allicin (LM: 0.42 mg/kg; allicin: 8 mg/kg), or PECm-Allicin@LM (LM: 0.42 mg/kg; allicin: 8 mg/kg). Blood samples were collected from the jugular vein at specified time points (before dosing and 3 min, 15 min, 30 min, 1 h, 2 h, 4 h, 6 h, 8 h, and 24 h after dosing). Each blood sample was centrifuged at 3000 rpm for 10 min at 4 °C to obtain serum. The serum samples were analyzed using liquid chromatography–tandem mass spectrometry (LC-MS/MS) and a noncompartmental analysis model of the Drug and Statistics (DAS) software (version 3.3.1, BioGuider Co., Shanghai, China) to assess the pharmacokinetics (PK) of LM.

A total of 132 specific pathogen-free (SPF) healthy male ICR mice (6–8-weeks-old; weight 22–25 g) were obtained from the Guangdong Medical Laboratory Animal Center. All animal experiments were approved by the Institutional Animal Care and Use Committee (IACUC) of the Institute of Science and Technology, Guangzhou University of Chinese Medicine (ISTGUCM). The mice were housed in the Laboratory Animal Room at the ISTGUCM, adhering to the guidelines for animal protection and welfare.

A total of 132 male ICR mice were randomly divided into six groups (*n* = 22); 10 mice were used to observe weight, infection rate, and survival cycle, and the other 12 were sampled on day 7 for a series of indicators. One group was intraperitoneally injected with PBS (Normal group), and the other five groups were intraperitoneally injected with 1 × 10^7^ freshly infected erythrocytes. Two hours after injection, mice in each of the five infected groups received an injection of PBS (Model group), LM (0.84 mg/kg), allicin (16 mg/kg), LM + Allicin (LM: 0.84 mg/kg; allicin: 16 mg/kg), or PECm-Allicin@LM (LM: 0.84 mg/kg; allicin: 16 mg/kg) into the tail vein. Over the next three days, the mice were administered the respective injection once a day, for a total of four treatments. The infection rate of *Pb*ANKA-infected mice was determined using the Giemsa stain kit on days 5 (day 1 of withdrawal), 6 (day 2 of withdrawal), 8 (day 4 of withdrawal), 10 (day 6 of withdrawal), and 12 (day 8 of withdrawal). Body weight was recorded on days 1, 3, 5, 7, 9, and 11. On day 7 of the experiment, blood samples were collected from 12 mice in each group after anesthesia for routine blood analysis (*n* = 6) and serum biochemistry (*n* = 6). The total number of red blood cells (RBCs) and hemoglobin (HGB) content in the whole blood samples were measured using an automatic blood cell counter (ADVIA2120i, Siemens, Germany). Alanine aminotransferase (ALT), aspartate aminotransferase (AST), creatinine (CRE), and urea (UREA) levels were measured using a fully automated biochemical analyzer (7180, HITACHI, Japan).

After blood collection, the mice were euthanized under anesthesia using cervical dislocation, and the major organs (heart, liver, spleen, lungs, kidneys, and brain) were rapidly excised and weighed to calculate the organ index (organ weight-to-body weight, %). Six fresh spleens were randomly selected from each group to analyze spleen immune cells using flow cytometry, and the remaining tissues were fixed in 4% paraformaldehyde and processed for histological examination of hematoxylin and eosin (H&E)-stained sections.

Each fresh spleen was washed and ground in pre-cooled PBS. The homogenate was filtered through a 200-mesh sieve, and the filtrate was collected and centrifuged at 3000 rpm for 5 min at 4 °C. The supernatant thus obtained was removed, and 1 mL of modified red blood cell lysis buffer (Meilunbio, China) was added to lyse the erythrocytes, in accordance with the manufacturer's instructions. After 10 min, an equal amount of PBS was added to terminate the lysis. The mixture was filtered through a 200-mesh sieve, and the filtrate was centrifuged to collect the cells. The cells were then washed with PBS and resuspended to obtain a splenic single-cell suspension. The suspended cells were incubated with anti-CD11c-EV450, anti-MHC II-PerCP-Cy5.5, and anti-PD-L1 (CD274)-PE or with anti-CD45-EV450, anti-CD3-ER780, anti-CD4-APC, anti-CD8-FITC, PD-1(CD274)-PE-Cy7, and anti-PD-L1(CD274)-PE, in accordance with the specifications for the respective antibodies. Subsequently, the expression of MHC II and PD-L1 on the surface of dendritic cells (DCs), CD4^+^ T cells, CD4^+^ PD-1^+^ T cells, CD4^+^ PD-L1^+^ T cells, CD8^+^ T cells, CD8^+^ PD-1^+^ T cells, and CD8^+^ PD-L1^+^ T cells was analyzed using flow cytometry.

All data were analyzed using the IBM SPSS Statistics 22.0 and are presented as mean ± standard error (SD) or mean ± standard error of the mean (SEM). According to the characteristics of the data, *one-way ANOVA*, *Welch's ANOVA*, the *Kruskal*–*Wallis test*, or the *Kaplan*–*Meier* test was used for statistical analysis. *P* < 0.05 was considered to indicate a statistically significant difference, and *P* < 0.01 was considered to indicate a statistically extremely significant difference.

## 3 Results

**Fig. 1:**
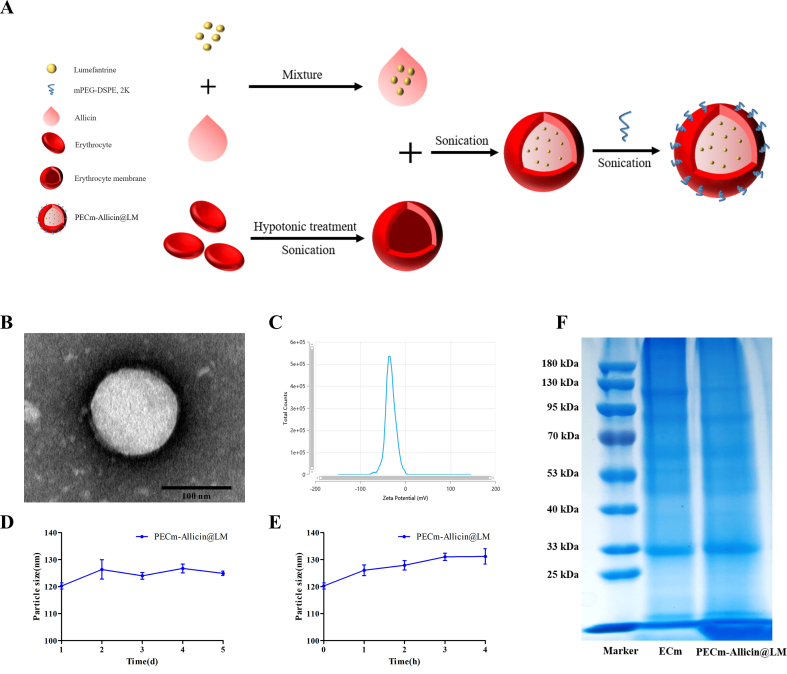
Synthesis and characterization of PECm-Allicin@LM. A Schematic of the process of PECm-Allicin@LM preparation. B TEM image of PECm-Allicin@LM. Scale bar: 100 nm. C Zeta potential of PECm-Allicin@LM. D Stability of PECm-Allicin@LM in 1× PBS at 4 °C, reflected by nanoparticle size over 5 days. E Stability of PECm-Allicin@LM in 10% FBS at 37 °C, reflected by nanoparticle size over 4 h. F SDS-PAGE profiling of protein composition. ECm stands for erythrocyte membrane. Data are presented as mean ± SEM (*n* = 3).

PECm-Allicin@LM was synthesized using the ultrasonic method applied in a previous study (Chen et al., 2019). PECm-Allicin@LM is a cell membrane-cloaked oil nanoparticle that has been modified with mDSPE-PEG_2000_ to make it more stable, and the allicin oil core contains LM, a water-insoluble antimalarial drug (Fig. 1A). The morphology of PECm-Allicin@LM was observed using TEM (JEM-1400plus). The spherical core–shell structure of PECm-Allicin@LM observed under TEM was consistent with an oil droplet enclosed in a membrane shell, indicating that an oil nanodroplet was successfully enclosed in the erythrocyte membrane (Fig. 1B). As shown in Table 1 and Fig. 1C, the zeta potential of PECm-Allicin@LM was −31.35 ± 2.02 mV. The particle size and PDI of PECm-Allicin@LM were 120.27 ± 1.96 nm and 0.169 ± 0.028, respectively (Table 1), and exhibited good stability at 4 °C for 5 days and even in 10% FBS at 37 °C for 4 h, with minor changes in size (Fig. 1D and Fig. 1E). SDS-PAGE showed that PECm-Allicin@LM retained almost all the major proteins on the erythrocyte membrane (Fig. 1F).

**Table 1:** Characterization of PECm-Allicin@LM.

		PECm-Allicin@LM
Particle size (nm)		120.27 ± 1.96
PDI		0.169 ± 0.028
Zeta potential (mV)		−31.35 ± 2.02
Encapsulation efficiency (%)	Lumefantrine	96.90 ± 0.22
	Allicin	80.74 ± 2.40
Drug loading (%)	Lumefantrine	3.52 ± 0.07
	Allicin	67.39 ± 0.65

Note: Data are expressed as mean ± SD (*n* = 3).

After the structure of PECm-Allicin@LM was determined, the encapsulation efficiency (%) and drug loading (%) of LM and allicin in the nanoparticles were investigated by HPLC. The results showed that the presence of erythrocyte membrane and mDSPE-PEG2000 did not affect the normal absorption of LM and allicin, indicating that the method has high specificity and could be used to determine the contents of LM and allicin after the nanoparticles are demulsified (Fig. S1). The encapsulation efficiency (%) and drug loading (%) of LM in PECm-Allicin@LM, as estimated using the HPLC method, were approximately 96.90 ± 0.22% and 3.52 ± 0.07%, respectively, and those of allicin were approximately 80.74 ± 2.40% and 67.39 ± 0.65%, respectively (Table 1 and Fig. S1). As shown in Fig. S2, the release curves of LM and PECm-Allicin@LM were in accordance with the first-order equation. PECm-Allicin@LM had a significantly slower release rate than free LM, indicating that PECm-Allicin@LM could achieve sustained release of LM.

**Fig. 2:**
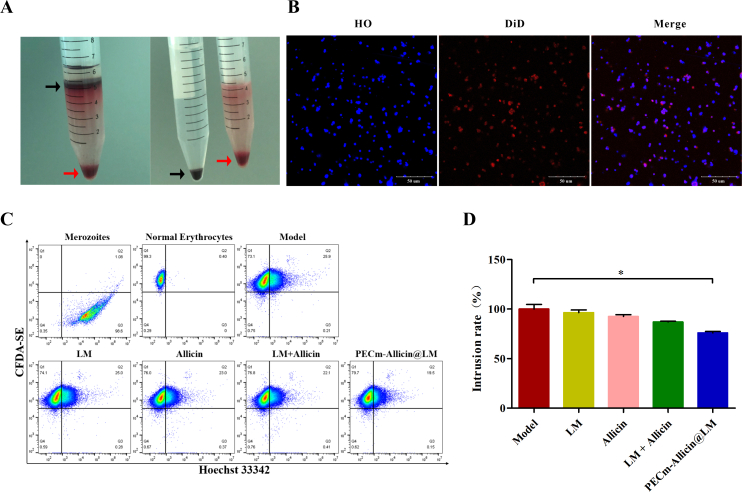
Neutralization of merozoites. A Purification of infected erythrocytes using the Percoll separation solution, displaying infected (black arrow) and normal (red arrow) erythrocytes. B Confocal fluorescent images of merozoites (blue), PECm-Allicin@LM (red), and their colocalization (purple). Scale bar = 50 μm. C Representative scatter plots of Hoechst 33342/CFDA-SE for the invasion test of merozoites and normal erythrocytes after drug treatment. D Intrusion rate (the percentage of cells in the Q2 area in each group relative to that in the Q2 area in the Model group). Data are presented as mean ± SEM (*n* = 3), * *P* < 0.05. (For interpretation of the references to colour in this figure legend, the reader is referred to the web version of this article.)

The Percoll solution precipitated normal erythrocytes to the bottom of the test tube and separated the infected erythrocytes into the upper layer (Fig. 2A, left). After the upper layer of infected erythrocytes was removed and centrifuged with 1× PBS washing, dark brown cells significantly differing in colour from the red normal erythrocytes, were precipitated (Fig. 2A, right). Using confocal laser scanning microscopy, we observed that DiD-labeled PECm-Allicin@LM (red fluorescence) bound to HO-labeled merozoites (blue fluorescence) and emitted purple fluorescence (Fig. 2B). To further investigate whether PECm-Allicin@LM could inhibit the invasion of normal erythrocytes by merozoites, we simulated the process *in vitro.* Normal erythrocytes were stained with CFDA-SE (Q1 area) and merozoites were stained with HO (Q3 area); the merozoite-infected erythrocytes were positive for both stains (Q2 area) (Fig. 2C). In this *in vitro* model, different treatments (PBS, LM, Allicin, LM + Allicin or PECm-Allicin@LM) produced different percentages of cells in the Q2 area, with the PECm-Allicin@LM group (PECm-Allicin@LM treatment) exhibiting a significantly lower percentage of cells in the Q2 area than that in the PBS group (Fig. 2C and Fig. 2D). For a better assessment of the impact of PECm-Allicin@LM on the intrusion rate, we set the intrusion rate for the Model group at 100%; the free drugs (LM, Allicin, LM + Allicin) had a slight effect on merozoite invasion, whereas the average intrusion rate for the PECm-Allicin@LM group was approximately 75.89%. This indicated that PECm-Allicin@LM inhibited merozoites from invading normal erythrocytes owing to the interaction between the erythrocyte membrane coating and merozoites (Fig. 2D).

**Fig. 3:**
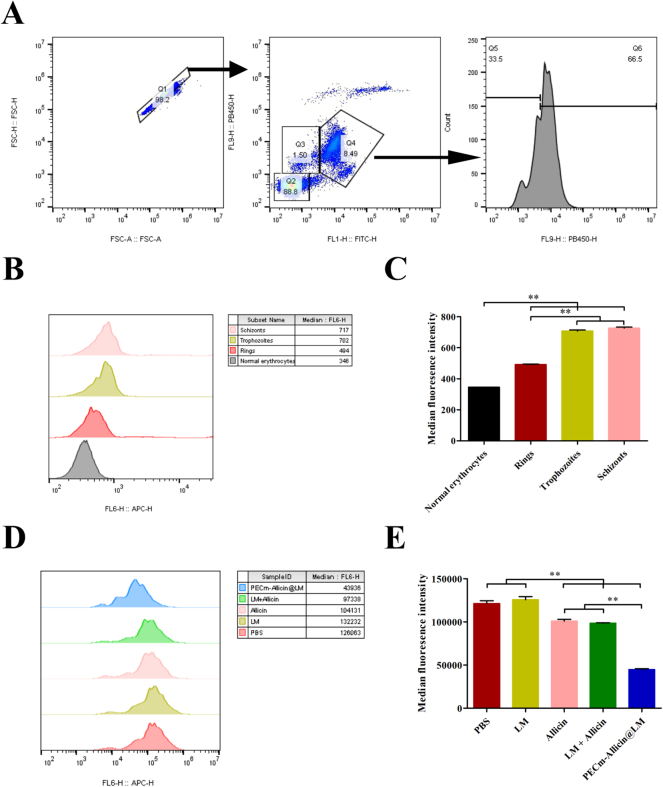
*In vitro* treatment of erythrocytes from experimental malaria mice with different drugs. A Analysis of the growth cycle of infected erythrocytes using the HO/TO double-staining method with flow cytometry. B Representative flow histograms for the intensity of DiD fluorescence from infected erythrocytes at different stages. C Intensity of DiD fluorescence from infected erythrocytes at different stages. D Representative flow histograms for the fluorescence intensity from mitochondria labeled with MitoTracker™ Deep Red FM Dye in infected erythrocytes subjected to different drug treatments. E Fluorescence intensity of mitochondria labeled with MitoTracker™ Deep Red FM Dye in infected erythrocytes subjected to different drug treatments. Data are presented as mean ± SEM (*n* = 3), **P < 0.01. (For interpretation of the references to colour in this figure legend, the reader is referred to the web version of this article.)

The HO/TO double-staining method was used to analyze the intracellular DNA/RNA levels in erythrocytes from experimental malaria mice, *via* flow cytometry, as previously reported (He et al., 2023). The erythrocytes from experimental malaria mice could be divided into normal (Q2, HO−/TO−) and infected (Q3 and Q4, HO+/TO− and HO+/ TO+) erythrocytes, where Q3 represents the rings and Q4 represented the trophozoites and schizonts (Fig. 3A). Based on the fluorescence intensity of DNA, Q4 was further divided into Q5 and Q6, representing trophozoites and schizonts, respectively (Fig. 3A). After incubating the erythrocytes from experimental malaria mice with PECm-Allicin@DiD, we found that PECm-Allicin@DiD was mainly distributed in schizonts and trophozoites, followed by rings, and was least distributed in normal erythrocytes, indicating that allicin-based biomimetic nanoparticles of the erythrocyte membrane can deliver drugs more effectively to infected erythrocytes (Fig. 3B−C).

The mitochondrial activity of infected erythrocytes treated with different drugs was detected using the MitoTracker™ Deep Red FM Dye. The fluorescence intensity emitted by the infected erythrocytes decreased upon treatment with PECm-Allicin@LM, LM + Allicin, and Allicin compared with that in the PBS and LM treatments, and PECm-Allicin@LM decreased the fluorescence intensity more efficiently than the other treatments (Fig. 3D−E). The fluorescence intensity indicates the mitochondrial activity of the infected erythrocytes, and it decreases when the *plasmodium* mitochondria are destroyed. These results therefore indicate that PECm-Allicin@LM decreased the *plasmodium* mitochondria more efficiently than the other drugs and that PECm-Allicin@LM killed the parasites *via* mitochondrial dysfunction.

**Fig. 4:**
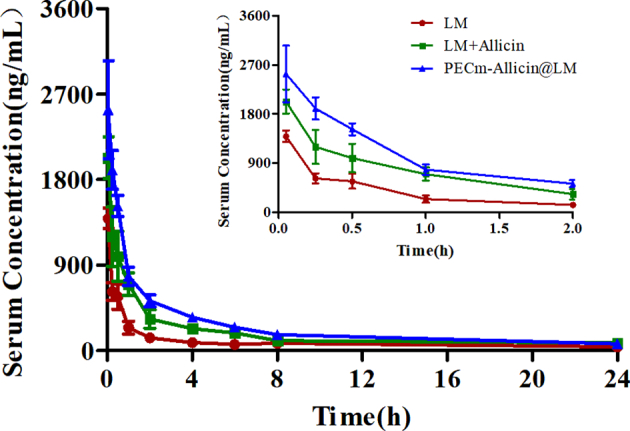
Mean serum concentration–time curve for lumefantrine (LM) in rats after a single intravenous injection of LM (0.42 mg/kg), LM + Allicin (LM: 0.42 mg/kg; allicin: 8 mg/kg), or PECm-Allicin@LM (LM: 0.42 mg/kg; allicin: 8 mg/kg), respectively.

We established a simple, rapid, and accurate LC-MS/MS method for the determination of LM in the serum of dosed rats. Typical chromatograms of rat blank serum, blank serum spiked with LM and the IS, and dosed rat serum samples are presented in Fig. S3. The mean serum concentration − time curves for LM in rats are shown in Fig. 4, and the PK parameters for LM, LM + Allicin, and PECm-Allicin@LM are listed in Table 2. After a single intravenous injection of LM (0.42 mg/kg), LM + Allicin (LM: 0.42 mg/kg; allicin: 8 mg/kg), or PECm-Allicin@LM (LM: 0.42 mg/kg; allicin: 8 mg/kg) to rats, LM was detectable from 3 min to 24 h. Although no statistical difference was noted between time to maximum serum concentration (C_max_) and terminal half-life (t_1/2_) among the three groups, compared with the LM group, the area under the concentration–time curve from time zero to the time of the last measurable serum concentration (AUC_0−t_) for LM in the PECm-Allicin@LM group was extremely significantly higher (*P* < 0.01), and the area under the concentration–time curve from time zero to infinity (AUC_0-∞_) for LM in the LM + Allicin and PECm-Allicin@LM groups was significantly higher (*P* < 0.05), which indicated that allicin could significantly improve the pharmacokinetic behavior of LM, such that *in vivo* exposure to LM could be increased. Moreover, the abovementioned effect was better when the free drugs were incorporated into PECm-Allicin@LM.

**Table 2:** Pharmacokinetic parameters for intravenously administered lumefantrine in rats.

Parameters	LM	LM + Allicin	PECm-Allicin@LM
AUC_0-t_ (h × ng/mL)	2065.40 ± 947.68	4431.40 ± 1089.85	6077.25 ± 609.79^⁎⁎^
AUC_0-∞_ (h × ng/mL)	2346.41 ± 849.03	6230.55 ± 1853.80^⁎^	7097.90 ± 593.68^⁎^
C_max_ (ng/mL)	1393.33 ± 182.30	2030.00 ± 381.58	2536.67 ± 895.79
t_1/2_ (h)	9.10 ± 4.12	14.42 ± 5.04	9.81 ± 1.03

Note: Data are expressed as mean ± SD (*n* = 3). *P < 0.05 significant difference *vs.* the LM group and **P < 0.01 extremely significant difference *vs.* the LM group.

**Fig. 5:**
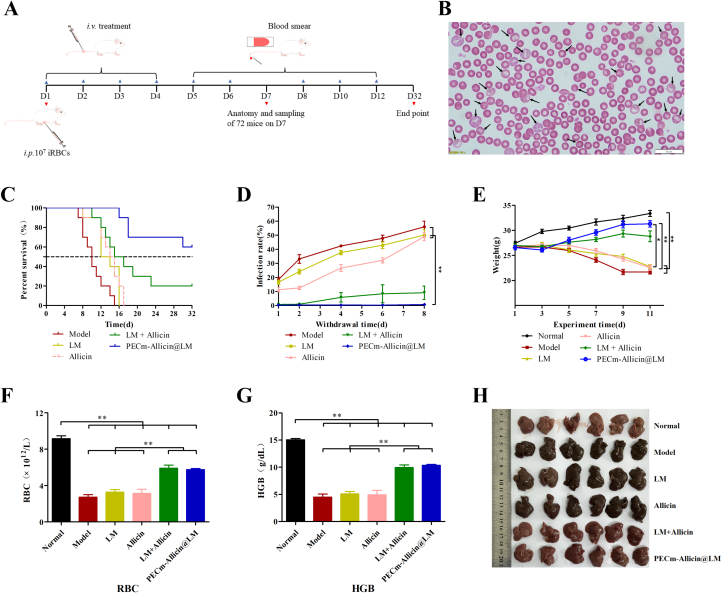
*In vivo* therapeutic effect of PECm-Allicin@LM. A Schematic of the treatment plan. B Peripheral blood smear subjected to Giemsa staining (*Pb*ANKA-infected erythrocytes marked with a black arrow). C Survival curves for *Pb*ANKA-infected ICR mice subjected to different treatments (*n* = 10). D Infection rate curves for *Pb*ANKA-infected ICR mice subjected to different treatments (*n* = 10). E Weight curves for ICR mice in different groups (*n* = 10). F Red blood cell count in peripheral blood samples from ICR mice in different groups (*n* = 6). G Hemoglobin concentration in peripheral blood samples from ICR mice in different groups (*n* = 6). H Appearance of livers from ICR mice in different groups. Data are presented as mean ± SEM, and * *P* < 0.05, ** *P* < 0.01. (For interpretation of the references to colour in this figure legend, the reader is referred to the web version of this article.)

To evaluate the antimalarial effects of PECm-Allicin@LM, ICR mice were used to establish an experimental malaria model. A schematic representation of the treatment plan is shown in Fig. 5A. *Pb*ANKA-infected erythrocytes were identified by Giemsa staining of blood smears (Fig. 5B). The indices for the evaluation of antimalarial effects, namely survival time, infection rate, body weight, anemia index, and liver morphology, were evaluated. Survival analysis showed that the median survival times for the LM, Allicin, and LM + Allicin groups were 12, 14, and 15 days, respectively, which were longer than those for the Model group (10 days) (Fig. 5C). All mice in the Model, LM, and Allicin groups reached humane clinical endpoints, but two mice in the LM + Allicin group and six in the PECm-Allicin@LM group were still alive (Fig. 5C), with the corresponding survival rates being 20% and 60%, respectively. Giemsa staining was performed to determine the infection rate in *Pb*ANKA-infected mice. As shown in Fig. S4 and Fig. 5D, the infection rate of peripheral blood cells increased with time after malaria infection (except for the PECm-Allicin@LM group). On day 8 of treatment, the infection rate of peripheral blood cells in the PECm-Allicin@LM group was significantly lower than those in the Model, LM, and Allicin groups. As shown in Fig. 5E, mice in the Model, LM, and Allicin groups gradually lost weight after infection. The body weights of mice in the PECm-Allicin@LM group were significantly higher than those in the LM and Allicin groups. After *Pb*ANKA infection, the erythrocyte count and hemoglobin concentration in the peripheral blood of mice decreased significantly compared with those in normal mice (Fig. 5F and G), indicating that malaria infection caused anemia. Both LM + Allicin and PECm-Allicin@LM alleviated anemia in *Pb*ANKA-infected mice to a greater extent than LM or Allicin (Fig. 5F and G). On day 7 of the experiment, some mice were dissected and their livers were excised. The livers of normal mice were brownish red, whereas those of *Pb*ANKA-infected mice were blackish brown, indicating a large amount of pigment deposition (Fig. 5H). LM or allicin treatment slightly alleviated malarial pigmentation in mouse livers, whereas LM + Allicin and PECm-Allicin@LM significantly reduced such pigmentation, and the appearance of the livers was approached that in normal mice (Fig. 5H).

In summary, PECm-Allicin@LM significantly reduced mortality, infection rate, and liver pigmentation and alleviated weight loss and anemia in *Pb*ANKA-infected mice, exhibiting better *in vivo* antimalarial effects than free drugs. The enhanced efficacy of PECm-Allicin@LM may be attributed to merozoites capture, targeted delivery, mitochondrial injury, and improved pharmacokinetics.

**Fig. 6:**
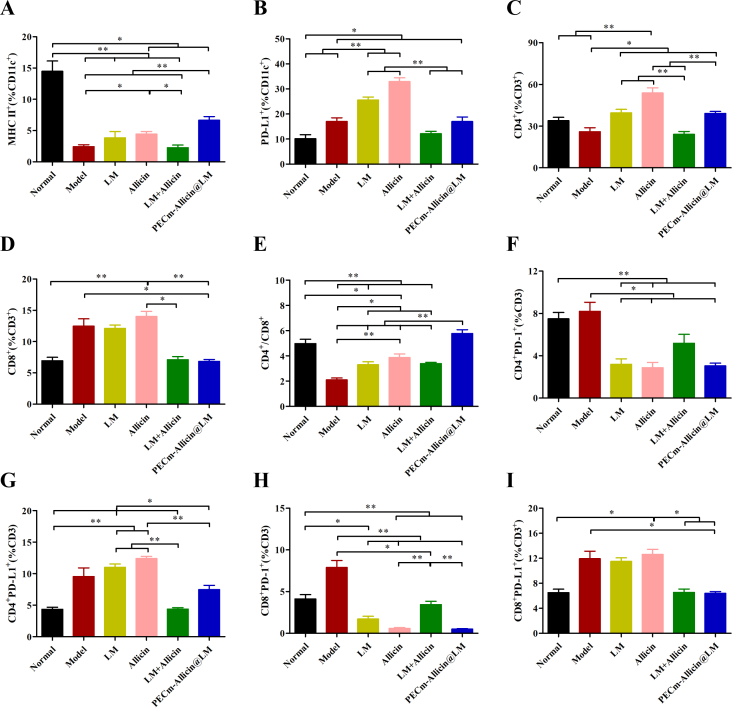
Immunomodulatory effect of PECm-Allicin@LM. Percentage of MHC II^+^ CD11c^+^ DCs (A), PD-L1^+^ CD11c^+^ DCs (B), CD4^+^ T cells (C). and CD8^+^ T cells (D). Ratio of CD4^+^ to CD8^+^ T cells (E). Percentage of PD-1^+^ CD4^+^ T cells (F), PD-L1^+^ CD4^+^ T cells (G), PD-1^+^ CD8^+^ T cells (H), and PD-L1^+^ CD8^+^ T cells (I). Data are presented as mean ± SEM (*n* = 6), * *P* < 0.05, ** *P* < 0.01.

To better understand the mechanism underlying the antimalarial effect of PECm-Allicin@LM, splenic immune cells were analyzed using flow cytometry (Fig. S5 and S6). As shown in Fig. S5B and Fig. 6A, the proportion of MHC II^+^ CD11c^+^ DCs in the spleen of PBS-treated *Pb*ANKA-infected mice was decreased compared with that in normal mice, and both allicin and PECm-Allicin@LM alleviated this decrease, with the latter being better. The proportion of PD-L1^+^ CD11c^+^ DCs cells in the spleen of PBS-treated *Pb*ANKA-infected mice was increased compared with that in normal mice, and both LM and allicin exacerbated this increase; however, LM + Allicin and PECm-Allicin@LM had no significant effect on this increase (Fig. S5C and Fig. 6B). LM and allicin increased the proportion of CD4^+^ T cells in the spleen of *Pb*ANKA-infected mice; PECm-Allicin@LM retained this regulatory effect, whereas LM + Allicin did not (Fig. S6B and 6C). PECm-Allicin@LM decreased the proportion of CD8^+^ T cells in the spleen of *Pb*ANKA-infected mice (Fig. S6B and 6D). The ratio of CD4^+^ to CD8^+^ T cells in the spleen of PBS-treated *Pb*ANKA-infected mice was decreased compared with that in normal mice. All four treatments alleviated this decline, and PECm-Allicin@LM performed better (Fig. S6B and 6E). Both LM and allicin decreased the proportion of PD-1^+^ CD4^+^ T cells in the spleen of *Pb*ANKA-infected mice; PECm-Allicin@LM retained this regulatory effect, whereas LM + Allicin did not (Fig. S6C and 6F). The proportion of PD-L1^+^ CD4^+^ T cells in the spleen of LM- or allicin-treated *Pb*ANKA-infected mice was increased compared with that in normal mice, whereas both LM + Allicin and PECm-Allicin@LM alleviated this increase (Fig. S6E and 6G). In all four treatments, the percentage of PD-1^+^ CD8^+^ T cells in the spleen of *Pb*ANKA-infected mice was decreased, and PECm-Allicin@LM also decreased the proportion of PD-L1^+^ CD8^+^ T cells (Fig. S6D and S6F and 6H − I).

In summary, *Pb*ANKA damaged the immune system of ICR mice; PECm-Allicin@LM could promote the maturation of spleen DCs, upregulate the ratio of CD4^+^ and CD8^+^ T cells, and downregulate the expression of immune checkpoint molecules (PD-1 and/or PD-L1) on the surface of DCs and T lymphocytes (including CD4^+^T and CD8^+^ T cells) to exert its immunomodulatory effects.

**Fig. 7:**
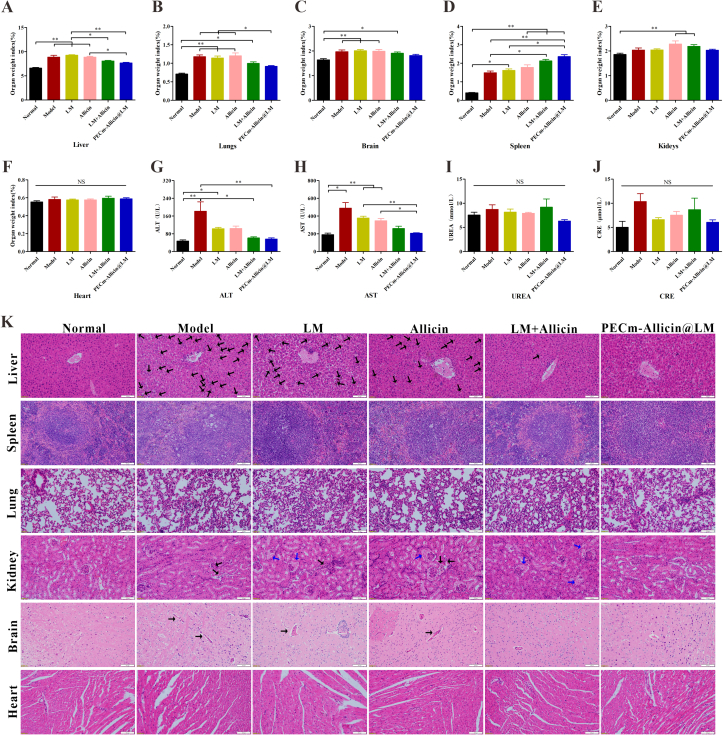
Safety assessment of PECm-Allicin@LM. A−F Organ indices for the liver, spleen, lungs, kidneys, brain, and heart (*n* = 12). G−H Analysis of liver function (ALT and AST, *n* = 6). I − J Analysis of renal function (UREA, CRE, *n* = 6). K H&*E*-stained slices of the liver, spleen, lung, kidney, brain, and heart. Scale bar = 100 μm. Hemozoin (black arrow) and abnormal exudate from the glomerular balloon cavity (blue arrow). Data are presented as mean ± SEM, * *P* < 0.05, ** *P* < 0.01; NS: *P* > 0.05. (For interpretation of the references to colour in this figure legend, the reader is referred to the web version of this article.)

The liver, lungs, and brain weight indices for mice in the Model group were significantly higher than those of mice in the normal group, indicating that *Pb*ANKA infection caused enlargement of these organs (Fig. 7A−C). PECm-Allicin@LM tended to inhibit this enlargement with the abovementioned weight indices for the PECm-Allicin@LM group not significantly different from those for normal mice (Fig. 7A−C), suggesting that PECm-Allicin@LM protected these organs in infected mice. The spleen weight indices for all drug treatment groups were significantly higher than those for normal mice; in particular, in the allicin-containing groups, the drugs did not appear to cause the spleen damage but most likely activated immune function in the spleen causing it to become enlarged (Fig. 7D). The kidneys weight index for the Allicin and LM + Allicin groups was significantly higher than that for the normal group, indicating that free allicin caused kidney enlargement (Fig. 7E). However, no statistical difference in the kidneys weight index was noted between the normal and PECm-Allicin@LM groups (Fig. 7E), suggesting that the preparation of allicin as nanoparticles prevented such changes in the kidney. No significant difference in the heart weight index was noted among the groups (Fig. 7F). The liver function indices (AST and ALT) for the Model group were significantly higher than those for the normal group, suggesting that *Pb*ANKA infection caused the liver damage (Fig. 7G−H). After treatment with PECm-Allicin@LM, the above indices for *Pb*ANKA-infected mice were not significantly different from those for normal mice (Fig. 7G−H), suggesting that PECm-Allicin@LM protected liver function in infected mice. No significant differences in the renal function indices (UREA and CRE) were evident among the groups (Fig. 7I−J).

H&E staining was used to assess histological alterations in different groups, including the liver, spleen, lung, kidney, and brain (Fig. 7K). Large amounts of hemozoin were visible in the liver and spleen in the Model group, and small amounts were observed in the kidney and brain; all four drugs alleviated the malarial pigmentation, with LM + Allicin and PECm-Allicin@LM performing better. In the kidneys from the LM, Allicin, and LM + Allicin groups, but not from the PECm-Allicin@LM group, an abnormal increase in exudate was observed in the glomerular balloon cavity (blue arrow), which might indicate drug-induced kidney injury. These results suggest that PECm-Allicin@LM is safer for intravenous administration than free drugs and has good biocompatibility.

## 4 Discussion

Nanotechnology has been used to explore new antimalarial treatments. It can help enrich the dosage forms of antimalarial drugs and enhance the effect of antimalarial therapy (Rahman et al., 2019). LM is an antimalarial drug that is slightly soluble in acetone and almost insoluble in ethanol and water, rendering its pharmaceutical development difficult. Moreover, LM is slowly absorbed. Only one oral dosage form of LM is used in clinical practice; the lack of intravenous administration restricts the clinical application of LM. In clinical application, artemether-LM combination therapy clears *Plasmodium* with different modes of action: LM interferes with the haem polymerization process, while artemether produces reactive oxygen species to kill *Plasmodium (*Dama et al., *2018**;* Antoine et al., *2014**)*. In this study, allicin-LM is a novel combination. Allicin inhibit *Plasmodium* growth in a dose-dependent way (Ounjaijean and Somsak, 2022). The antimalarial effect of allicin is related to the inhibition of proteolytic cleavage of circumsporozoite protein and cysteine protease (Waag et al., 2010; Ounjaijean and Somsak, 2022). Moreover, the immune regulation by allicin is beneficial for the antimalarial efficacy of the drug (Feng et al., 2012). The complementary antimalarial effects of allicin-LM showed definite potential in this study.

Zhang et al. reported a biomimetic oil nanosponge consisting of an olive oil nanodroplet wrapped in the erythrocyte membrane (Chen et al., 2019), which prompted us to prepare PECm-Allicin@LM. Previously, we discovered that allicin oil is capable of dissolving LM. In this study, we prepared PECm-Allicin@LM, an erythrocyte membrane-cloaked allicin oil nanoparticle modified with mDSPE-PEG_2000_ to make it more stable; the allicin oil core of this nanoparticle dissolved LM. DSPE-PEG is a biocompatible and amphiphilic material, important for the preparation of nanocarriers to achieve specific functions, such as improved stability, enhanced encapsulation efficiency, and prolonged blood circulation time (Che et al., 2015). PECm-Allicin@LM exhibits a moderate particle size, uniform PDI, and good stability; these attributes make it suitable for intravenous administration. Compared with oral administration, intravenous administration allows antimalarial drugs to act directly on erythrocytes infected with malaria parasites and quickly kill them, thereby helping save patients with severe malaria (Matshe et al., 2023; Plewes et al., 2019; Boateng-Marfo et al., 2021; Zeng et al., 2022).

We found that allicin can significantly improve the pharmacokinetic behavior of LM, and the abovementioned effects were better when the free drugs were prepared as PECm-Allicin@LM. Allicin can dissolve and load LM, and PECm-Allicin@LM enables LM to maintain a higher blood concentration and increase *in vivo* exposure owing to mDSPE-PEG_2000_, which results in LM action against malaria parasites in the blood being more efficient.

During the blood stage of malaria infection, merozoites in the blood invade normal erythrocytes by binding to specific sites on the surface of erythrocyte membranes (Molina-Franky et al., 2022). PECm-Allicin@LM is composed of oil nanodroplets encapsulated by the erythrocyte membrane. The SDS-PAGE results indicated that almost all the major proteins on the erythrocyte membrane were retained in PECm-Allicin@LM, which is beneficial for capturing merozoites and preventing them from invading normal erythrocytes in the blood. We referred to a previous study (He et al., 2023) and extracted *in vivo* merozoites and normal erythrocytes to briefly simulate the process of merozoites invading normal erythrocytes. Then, we verified through confocal laser scanning microscopy and flow cytometry whether the nanoparticles could capture merozoites and competitively inhibit infection. *In vitro* experiments showed that biomimetic erythrocyte membrane nanoparticles, PECm-Allicin@LM, can bind to merozoites and reduce their invasion of normal erythrocytes, which is consistent with previous reports (He et al., 2023; Najer et al., 2014). However, this is only a preliminary verification. Further verification requires more microscopic evidence, such as collecting dynamic quantitative evidence using *in-situ* transmission electron microscopy.

Some researchers have been dedicated to exploring strategies for targeted delivery of antimalarial drugs to infected erythrocytes, among which have wrapped nanoparticles in the erythrocyte membrane to target *Plasmodium*-infected erythrocytes *via* the “rose-wreath” effect (Najer et al., 2014; Borgheti-Cardoso et al., 2020). We also found that the PECm-Allicin@DiD is more likely to accumulate in *Pb*ANKA-infected erythrocytes than in normal erythrocytes, especially in schizonts and trophozoites. This effect allows LM, a schizonticide, to exert its antimalarial effects more accurately.

Although energy metabolism in *Plasmodium* parasites mainly occurs *via* glycolysis during the blood phase, the mitochondrial electron transport chain is also necessary, and complex III of the mitochondrial electron transport chain of *Plasmodium* parasites is the target of the antimalarial drug atovaquone (Vaidya and Mather, 2009; Koumpoura et al., 2021). The results of *in vitro* experiments suggested that allicin could rapidly act on *Pb*ANKA-infected erythrocytes and attack the mitochondria in *Pb*ANKA, destroying their function, which may be one of the main mechanisms for the antimalarial activity of allicin. LM had no significant effect on the mitochondria in *Pb*ANKA-infected erythrocytes *in vitro*, which may be related to its poor solubility; alternatively, the antimalarial mechanism of LM might not involve mitochondrial function. PECm-Allicin@LM significantly enhanced the inhibition of mitochondrial function, possibly because small and stable nanoparticles could enter infected erythrocytes more easily than free allicin.

Based on the results of the pharmacokinetic analyses, allicin can improve the pharmacokinetic behavior of LM, with a higher AUC, which means that it can increase the *in vivo* exposure of LM. Moreover, the improvement effect was more pronounced after the preparation of the free drugs into nanoparticles. The pharmacokinetics of nanomedicine is usually better than that of microsized drugs; as the particle size decreases, the efficacy of the drug in curing the disease increases (Ravindran et al., 2018). As reported, erythrocyte membrane (EM) was used as a biomimetic nanocoating for prolonged circulation time and reduced accelerated blood clearance. This is related to the functional components on EM. CD47 on the EM coated nanoparticles surface can be specifically recognized by SIRP-α on macrophages and activates the “don't eat me” signaling pathway to inhibit phagocytosis (Oldenborg et al., 2000; Hu et al., 2011). Moreover, EM-coated nanoparticles did not increase serum IgG and IgM levels (Rao et al., 2015). Compared with PEGylated nanoparticles, EM-coated nanoparticles exhibit a longer blood circulation time (Hu et al., 2011). Thus, EM coating can reduce macrophage clearance and prolong circulation. However, in our study, PECm-Allicin@LM did not prolong the C_max_ and t_1/2_ of LM. This might be related to the short duration of our PK experiments. Extending the experimental duration, such as to 48 or 72 h, can better evaluate whether PECm-Allicin@LM has a long circulation effect. The higher AUC_0-t_ and AUC_0-∞_ of PECm-Allicin@LM indicated that a higher concentration of LM existed in blood, which was beneficial for enhancing therapeutic efficacy.

Both LM and allicin showed *in vivo* antimalarial effects, but PECm-Allicin@LM was superior to the free drugs. Allicin has been reported to exhibit potential antimalarial activity alone as well as in combination with an antimalarial drug (artesunate) (Ounjaijean and Somsak, 2022; Coppi et al., 2006). In PECm-Allicin@LM, allicin not only dissolved and loaded LM but also enhanced the antimalarial effect, indicating that allicin/LM is an ideal drug combination. In addition, PECm-Allicin@LM improved the water solubility of LM, reduced the invasion of merozoites into normal erythrocytes, increased the accumulation of drugs in *Plasmodium*-infected erythrocytes, inhibited the mitochondrial activity of *Plasmodium* in the erythrocytic stage, and improved the pharmacokinetic behavior of LM, leading to superior antimalarial activity. With the emergence of drug-resistant cancers, new treatment methods to overcome drug resistance have gradually come into being, such as nanoparticle-based combination therapy (Wen et al., 2023; Wang et al., 2023; Hu and Zhang, 2012). Nanoparticles capable of multimodal treatment are increasing, not only in cancer but also in infectious diseases (Liping et al., 2023; Wang et al., 2024; Tonbul et al., 2024). For example, heparin-functionalized liposomes delivered primaquine selectively to infected erythrocytes (Marques et al., 2014). EM-coated nanoparticles exhibited targeting effects and enhanced antimalarial efficacy (Zuo et al., 2022). Compared with those delivery systems, PECm-Allicin@LM co-delivered lumefantrine and allicin to infected erythrocytes, trapped merozoites, and regulated immunity. It provides multimodal antimalarial treatment and has potential advantages. In this platform, LM was dissolved in the oil phase (allicin) and coated with EM by ultrasound. The manufacturing processes is simple and feasible. Many drugs, such as artemether (anti-malarial drug), moxifloxacin (anti-bacterial drug), paclitaxel (anti-tumor drug) and others, were hydrophobic; they could be dissolved in oil phase, and loaded into the EM-coated nanoparticles. Thus, this platform has the potential to be applied to other diseases.

Although some studies have reported that allicin has antimalarial effects, there are not many reports on its antimalarial mechanism. Gomes ARQ et al. proposed that supplementing antioxidants would help reduce the cellular damage caused by malaria to the host (Gomes et al., 2022). Allicin is an antioxidant with the ability to resist oxidative stress (Okada et al., 2006). Therefore, the antimalarial mechanism of allicin may be related to its antioxidant stress resistance, which requires further experimental exploration. Allicin reportedly partially protects the host against *P. yoelii* 17XL through enhancement of the production of pro-inflammatory mediators such as IFN-γ, TNF, IL-12p70 and NO, and promotion of the maturation of CD11c^+^ DCs (Feng et al., 2012). In our study, we found that the proportion of MHC II^+^ CD11c^+^ DCs and the ratio of CD4^+^ to CD8^+^ T cells in the spleen of PBS-treated PbANKA-infected mice were decreased compared with those in normal mice, allicin increased the proportion of MHC II^+^ CD11c^+^ DCs and the ratio of CD4^+^ to CD8^+^ T cells in the spleen of *Pb*ANKA-infected mice. Moreover, allicin significantly downregulated the increased PD-1 levels of CD4^+^ and CD8^+^ cells induced by malaria infection. These findings suggest that, in addition to destroying the mitochondria of the malaria parasite as mentioned above, promoting DC maturation and regulating T-cell immunity are also immune mechanisms underlying the antimalarial effects of allicin.

Malaria induces immune dysfunction. Mature DCs (MHC II^+^) play a pivotal role in initiating adaptive immune responses. In malaria-infected mice, DCs exhibited an immature phenotype with lower levels of MHC class II, which resulted from the malarial pigment (Skorokhod et al., 2004). Allicin could partially protect the host against malaria infection by enhancing the host immune response and promoting the maturation of DCs (Feng et al., 2012). PECm-Allicin@LM partially reversed the malaria infection-induced downregulation of DC maturation. Notably, monotherapy with LM or allicin increased PD-L1 expression in DCs. PD-L1 present on DCs plays an important role in inhibiting T-cell activation (Peng et al., 2020).The combination therapy (LM + Allicin or PECm-Allicin@LM) reduced the abundance of PD-L1^+^ DCs, which may help to revive the immune response.

Throughout infection, T cells determine the immunity and pathogenesis of malaria. The CD4^+^/CD8^+^ ratio in the Model group was lower than that in the normal group, indicating malaria infection-induced immune dysfunction. PECm-Allicin@LM increased the CD4^+^/CD8^+^ ratio to normal levels. The PD-1/PD-L1 immune checkpoints are prominent therapeutic targets in various diseases. PD-1 on CD8^+^ and CD4^+^ T cells mediates potent inhibitory signals that induce T-cell dysfunction, which leads to weak immune protection against malaria (Horne-Debets et al., 2013). PD-1 downregulation (PD-1 knockout or antibody-mediated) decreases the infection rate, accelerates parasite clearance, and prolongs survival, and is a novel strategy for malaria treatment (Horne-Debets et al., 2013; Pan et al., 2020). In this study, malaria infection tended to enhance the expression of PD-1 in T cells, whereas PECm-Allicin@LM downregulated PD-1 levels in T cells (CD4^+^ and CD8^+^) in infected mice. Notably, PD-L1 is also expressed on T cells besides tumor cells. Owing to the interaction between PD-1 and PD-L1, PD-L1^+^ T cells inhibit PD-1^+^ effector T-cells activity. Conversely, PD-L1^+^ T cells negatively regulate the immune responses of PD-1^+^ cells (Hu et al., 2024). Malarial infection also tended to enhance the expression of PD-L1 in T cells, and PECm-Allicin@LM exhibited a reciprocal effect, reducing PD-L1 levels in T cells (CD4^+^ and CD8^+^) in infected mice. The reduction in PD-1 and PD-L1 levels was hypothesized to contribute to the antimalarial efficacy observed in this study. Although research on the immune mechanism of antimalarial drugs is rather scarce, the immune response is important in both the stages of infection as well as in the treatment of malaria (Cai et al., 2020; Pohl and Cockburn, 2022). Furthermore, PECm-Allicin@LM showed good biocompatibility with no obvious damage to the major organs. Overall, our study indicated that PECm-Allicin@LM is a safe and effective strategy for targeting multiple pathways in malaria treatment.

The antimalarial effect of PECm-Allicin@LM is superior to that of free drugs. However, as previously reported (Xia et al., 2019), the limitations of bionic nanoparticles do exist, and PECm-Allicin@LM is no exception. As PECm-Allicin@LM is composed of biological material, one major limitation is achieving acceptable batch-to-batch variations, this is also a challenge for the scalability and formulation stability of PECm-Allicin@LM. Another major limitation is the quality control of PECm-Allicin@LM. It is crucial to ensure the absence of pyrogen contamination in erythrocyte membranes and to remove nanoparticles containing denatured proteins, in order to prevent potential immune responses triggered by endogenous antigens. In addition, it is necessary to ensure that biomimetic nanoparticles of erythrocyte membranes maintain structural integrity and functional activity during storage and *in vivo* circulation; this is another challenge for the scalability and formulation stability of PECm-Allicin@LM. Currently, there is no effective solution to the above problems. Precise control of particle size distribution, drug loading and polydispersity during the manufacturing process might be a feasible strategy to address variability. Exploring biomimetic membrane materials that can be synthesized to replace erythrocyte membranes and optimizing the preparation process can accelerate their translation into clinical treatment. Nonetheless, PECm-Allicin@LM offers a distinctive conceptual framework for the development of novel antimalarial therapeutics. Although allicin in PECm-Allicin@LM can achieve two goals at once, serving both as a nanomaterial and an antimalarial drug, it has been reported that its stability in blood is poor, as only traces of allicin could be detected after it was incubated in blood for 5 min (Freeman and Kodera, 1995). However, with the disappearance of allicin, diallyl disulfide emerged, which is relatively stable in the blood. The pros and cons of this change are unknown and require further evaluation in the future. Lastly, this study was performed in mice; there are still many challenges to overcome in the preparation and application of PECm-Allicin@LM from murine to human malaria models. Humanized mouse model of malaria may provide a guide for antimalarial drug development with clinical potential (Minkah et al., 2018).

## 5 Conclusions

We developed PECm-Allicin@LM, an allicin-based biomimetic nanoparticle of erythrocyte membrane for the delivery of lumefantrine (LM), which showed excellent performance, suitable particle size, and good stability. PECm-Allicin@LM nanoparticles could deliver LM to *Plasmodium*-infected erythrocytes to kill the parasite and disguise themselves as erythrocytes to trap merozoites and competitively inhibit them from repeatedly infecting normal erythrocytes. Allicin in PECm-Allicin@LM not only dissolved LM and significantly improved its pharmacokinetic behavior but also synergistically protected against malaria and regulated immunity. Allicin plays the role of “killing two birds with one stone” as both a drug and an adjuvant. PECm-Allicin@LM improved antimalarial activity in mice, with no obvious organ toxicity, and thus may be useful for antimalarial applications.

## CRediT authorship contribution statement

**Chuyi Yu:** Writing – original draft, Investigation. **Xiaobo Li:** Investigation, Funding acquisition. **Keneng Cai:** Investigation. **Weichi Jiang:** Investigation. **Wanying Chen:** Investigation. **Run Xia:** Investigation. **Mengyao Xu:** Investigation. **Jianjia Feng:** Investigation. **Chengli Ling:** Investigation. **Sheng Zhou:** Investigation. **Yinhuan Chen:** Investigation. **Feng Zeng:** Investigation. **Qin Xu:** Investigation. **Xiao He:** Investigation. **Mingqiang Li:** Investigation. **Jianping Song:** Investigation, Funding acquisition. **Jianming Liang:** Writing – review & editing, Investigation, Funding acquisition, Conceptualization.

## Consent for publication

All authors agree with the submission and publication of this paper.

## Ethics approval and consent to participate

All animal experiments were approved by the Ethics Committee of Science and Technology Industrial Park, Guangzhou University of Chinese Medicine (PZ23025). All animal care protocols and experiments involved in this study were in compliance with institutional animal ethics guidelines.

## Funding

This work was supported by 10.13039/501100001809Natural Science Foundation of China (82204628, 82374315, 81873218), the Special Projects in Key Areas of Colleges and Universities in Guangdong Province (2022ZDZX2015), 10.13039/501100003453Natural Science Foundation of Guangdong Province (2022A1515011312), the Science and Technology Program of Guangzhou (2024A04J4899, 202206010066), Young top talent (team) cultivation of the “Unmasking” project (A1-2601-24-414-110Z76), Training Youth Engineering Discipline Talent Cultivation Project for Young Professionals (A1-2601-25-415-206Z578), the 10.13039/501100002858China Postdoctoral Science Foundation (2023M730808) and 10.13039/501100012166National Key Research and Development Program of China (2024YFC2310902).

## Declaration of competing interest

All authors declare no conflicts of interest.

## Data Availability

All data analyzed during this study are included in this article.

## Appendix A Supplementary data

Supplementary material Representative peak diagram of LM and allicin, representative chromatograms for LM and internal standard in rat serum, *in vitro* release, peripheral blood smears subjected to Giemsa staining, immunomodulatory effect of PECm-Allicin@LM on DCs and T cells. Image 1
