# Supplementary material for: Allicin-based biomimetic nanoparticles of the erythrocyte membrane for the delivery of lumefantrine to enhance its antimalarial effect
Source: Int J Pharm X. 2026 Jan 11;11:100487. doi: 10.1016/j.ijpx.2026.100487 (PMC13049907; doi:10.1016/j.ijpx.2026.100487)
Supplement: Supplementary file 1 — Supplementary material Representative peak diagram of LM and allicin, representative chromatograms for LM and internal standard in rat serum, in vitro release, peripheral blood smears subjected to Giemsa staining, immunomodulatory effect of PECm-Allicin@LM on DCs and T cells. [file mmc1.pdf]

Allicin-Based Biomimetic Nanoparticles of the Erythrocyte Membrane for the Delivery of Lumefantrine to Enhance Its Antimalarial Effect

Chuyi Yu[1,2], Xiaobo Li[1,3], Keneng Cai[1], Weichi Jiang[1,2,5], Wanying Chen[1], Run Xia[1], Mengyao Xu[1], Jianjia Feng[1],[2] Chengli Ling[4], Sheng Zhou[1], Yinhuan Chen[2], Feng Zeng[1], Qin Xu[1], Xiao He[1], Mingqiang Li[1], Jianping Song[1,2] and Jianming Liang*[1,2]

[1]Artemisinin Research Center, Guangzhou University of Chinese Medicine, Guangzhou 510006, China.

[2]The First Afffliated Hospital of Guangzhou University of Chinese Medicine, Guangzhou 510405, China.

[3]Qingyuan Polytechnic, Panlong Garden, Dongcheng Street, Qingcheng District, Qingyuan, Guangdong Province 511510, China.

[4]Hunan academy of Chinese Medicine, Yuehua road, Yuelu district, Changsha 410013, China

[5]Guangdong Provincial Second Hospital of Traditional Chinese Medicine, Guangzhou 510006, China.

Corresponding author:

Name: Jianming Liang, E-mail: liangjianming@gzucm.edu.cn.

HPLC analysis

The standard solution of allicin was detected by HPLC. As shown in Figure S1A, when the wavelength was 335 nm, LM appeared at 2.578 min. As shown in Figure S1B, the main peak of LM in PECm-Allicin@LM appeared at 2.570 min, similar to the standard LM. As shown in Figure S1C, when the wavelength was 214 nm, multiple main peaks of allicin appeared at 3.307, 4.083, 5.063, and 6.669 min. As shown in Figure S1D, the main peaks of allicin in PECm-Allicin@LM appeared at 3.342, 4.124, 5.111, and 6.719 min, similar to those of the standard allicin. The above results indicate that LM and allicin remain in PECm-Allicin@LM.

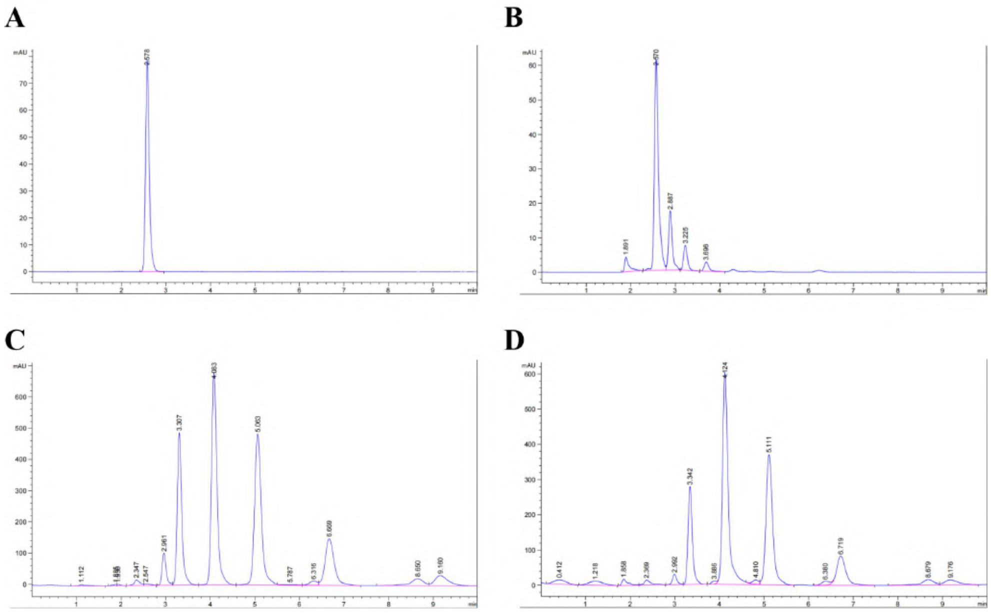

Figure S1 Construction of the HPLC conditions for LM and allicin. Representative peak diagrams of the LM standard product (**A**), LM in PECm-Allicin@LM (**B**), allicin standard product (**C**), allicin in PECm-Allicin@LM (**D**).

In Vitro **Release**

In vitro release of LM from the drugs (LM and PECm-Allicin@LM) was conducted by dialysis in a dialysis bag (14000 MW cut off) with 50 mL of release buffer solution (0.01 mol/L HCl, 5% 2-Hydroxypropyl-β-cyclodextrin) at 37 °C, respectively. The samples were agitated at 100 rpm, and the release medium (1 mL) was removed for analysis at given time intervals (0, 0.5, 1, 2, 4, 6, 8, 12, and 24 h), and replaced with the same volume of fresh release medium. The absorbance of the samples at 335 nm were measured using a Microplate Reader.

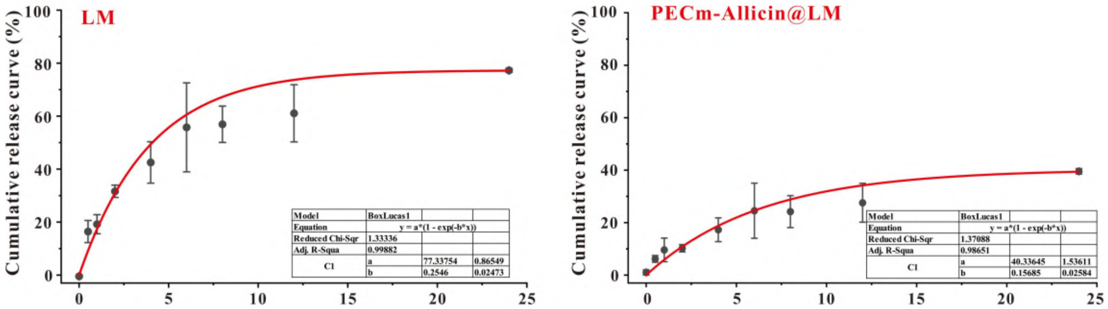

Figure S2 *In vitro* release curve of LM and PECm-Allicin@LM.

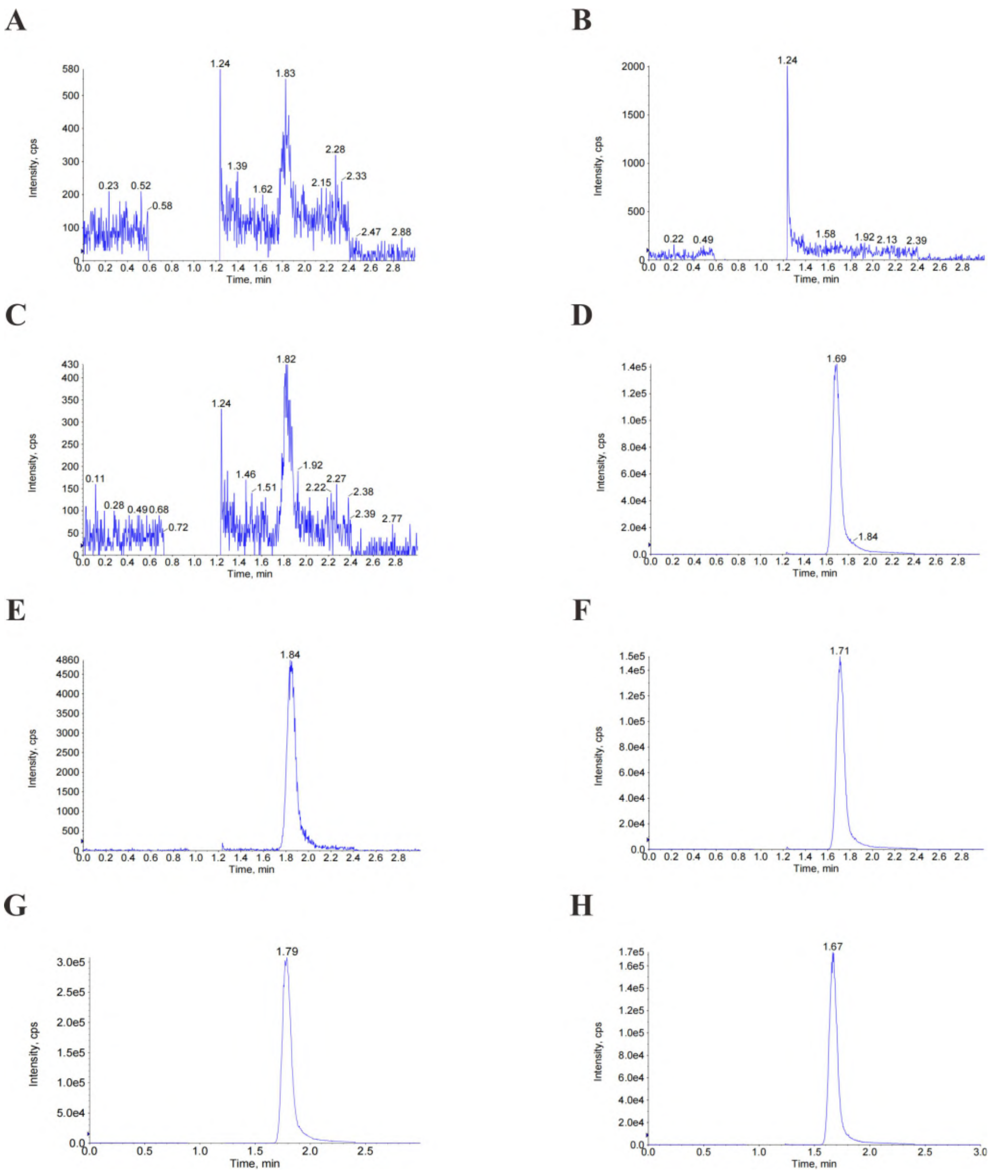

Figure S3 Representative chromatograms for LM and the internal standard (IS) (Lumefantrine-d9) in rat serum (AB Sciex QTRAP® 5500 LC/MS/MS). **A** Blank serum sample (LM). **B** Blank serum sample (IS). **C** Blank serum sample spiked with IS (LM). **D** Blank serum sample spiked with IS (IS). **E** Blank serum sample spiked with IS and LM (LM). **F** Blank serum sample spiked with IS and LM (IS). **G** Rat serum sample 30 min after the intravenous dose of PECm-Allicin@LM (LM). **H** Rat serum sample 30 min after the intravenous dose of PECm-Allicin@LM (IS).

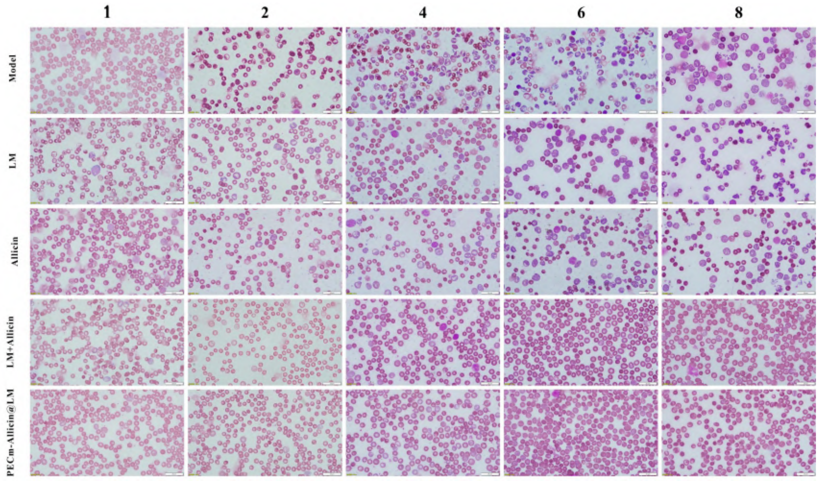

Figure S4 Peripheral blood smears subjected to Giemsa staining on days 5 (day 1 of withdrawal), 6 (day 2 of withdrawal), 8 (day 4 of withdrawal), 10 (day 6 of withdrawal), and 12 (day 8 of withdrawal) for *Pb*ANKA-infected ICR mice subjected to different treatments (n = 10).

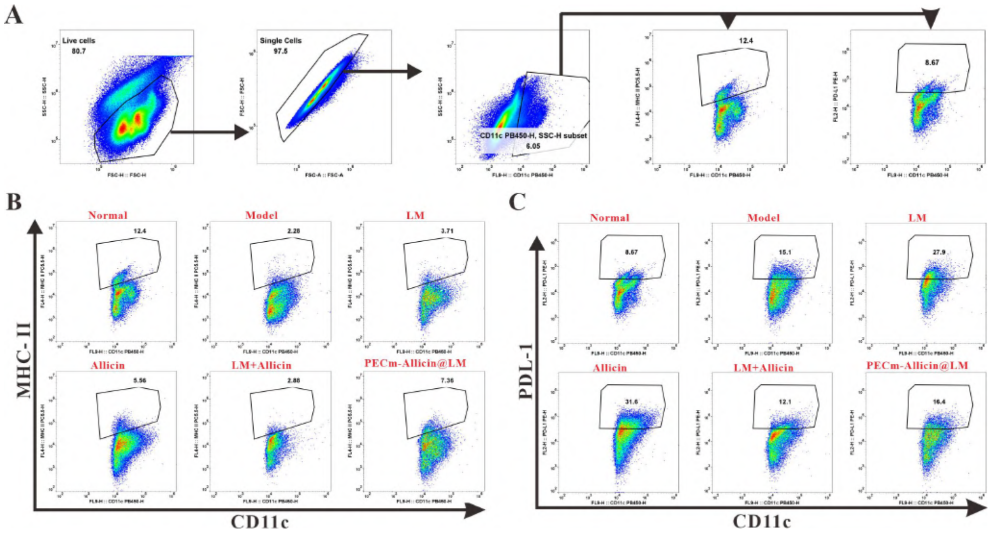

Figure S5 Immunomodulatory effect of PECm-Allicin@LM on DCs. Representative scatter plots of DCs ($CD11c^+$) (**A**), MHC II^+ $CD11c^+$ DCs (**B**), and PD-$L1^+$ $CD11c^+$ DCs (**C**).

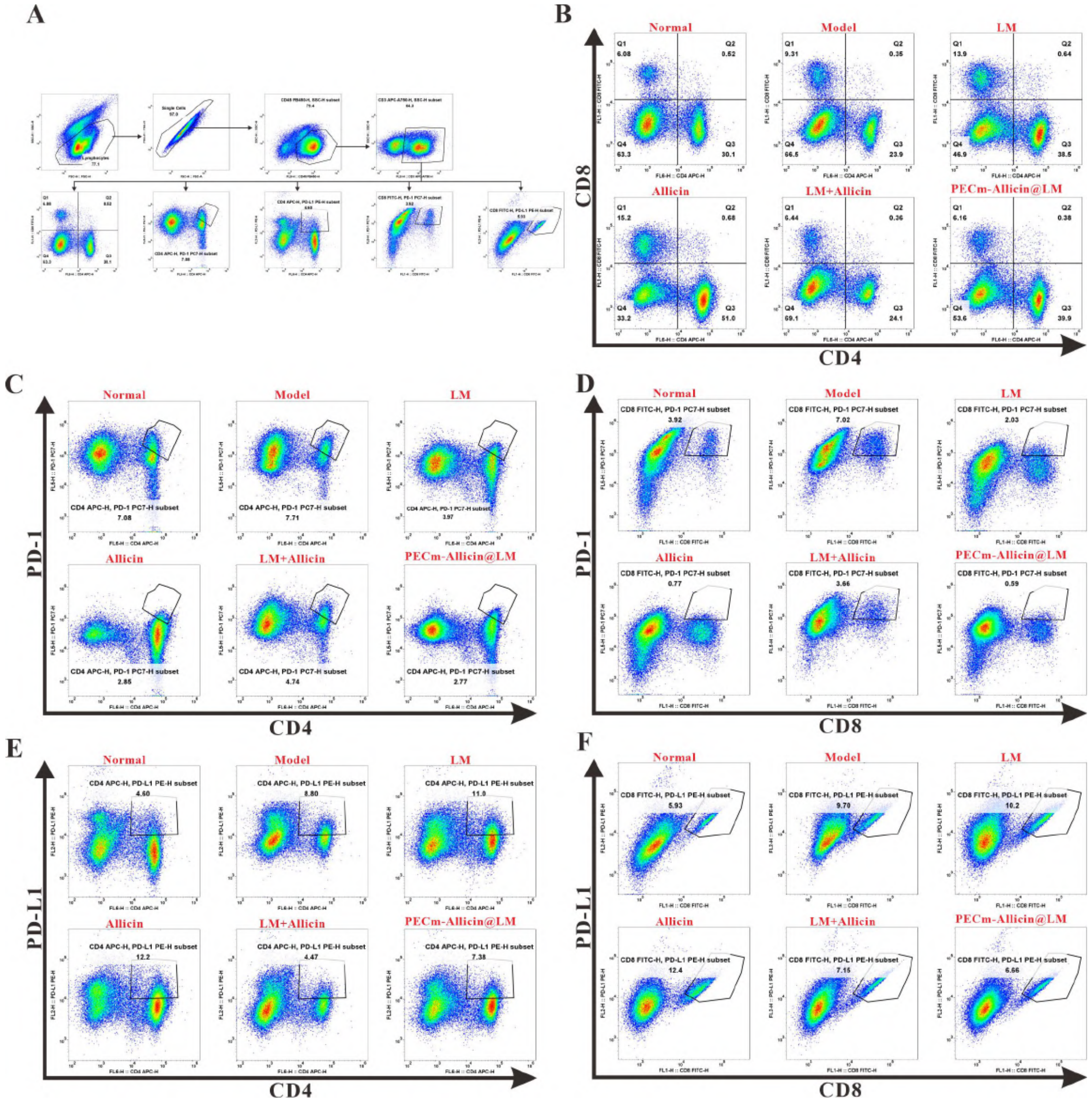

Figure S6 Immunomodulatory effect of PECm-Allicin@LM on T cells. Representative scatter plots of T cells (**A**), $CD4^+$ and $CD8^+$ T cells (**B**), $PD\text{-}1^+$ $CD4^+$ T cells (**C**), $PD\text{-}1^+$ $CD8^+$ T cells (**D**), $PD\text{-}L1^+$ $CD4^+$ T cells (**E**), and $PD\text{-}L1^+$ $CD8^+$ T cells (**F**).
